# Evaluating the effect of the COVID-19 pandemic on medical waste disposal using preference selection index with CRADIS in a fuzzy environment

**DOI:** 10.1016/j.heliyon.2024.e26997

**Published:** 2024-03-01

**Authors:** Ahmet Turan Demir, Sarbast Moslem

**Affiliations:** a Department of Biomaterials and Tissue Engineering, Institute of Graduate Studies, Tokat Gaziosmanpaşa University, 60250, Tokat, Turkey; b School of Architecture Planning and Environmental Policy, University College Dublin, Belfield, Dublin 15, D04 V1W8, Ireland

**Keywords:** Medical waste disposal, Preference selection index, Multi-criteria decision-making, CRADIS, Fuzzy sets, Sensitivity analysis

## Abstract

The COVID-19 pandemic has caused a surge in essential medical supplies usage, leading to a notable increase in medical waste generation. Consequently, extensive research has focused on sustainable disposal methods to handle used medical equipment safely. Given the necessity to evaluate these methods considering qualitative and quantitative criteria, this falls within the realm of multi-criteria decision-making (MCDM). This study introduces a framework for selecting the most suitable medical waste treatment methods, taking into account economic, technological, environmental, and social aspects. Sixteen criteria were assessed using the Fuzzy Preference Selection Index (F–PSI) to determine the optimal waste disposal approach. Additionally, the Fuzzy Compromise Ranking of Alternatives from Distance to Ideal Solution (F-CRADIS) method was employed to evaluate nine technologies for medical waste disposal. Notably, disinfection efficiency emerged as the most crucial criterion, with autoclaving identified as the preferred method for medical waste treatment. A practical case study conducted in Sivas, Turkey, validates the feasibility of these strategies. Multiple sensitivity analyses were performed to ensure the stability and reliability of the proposed approach.

## 1 Introduction

Medical waste, known as “health care waste” according to the World Health Organization (WHO) and the European Union (EU), and termed “infectious waste” in Japan, presents a significant challenge across various countries, including the United States, South Korea, and China [1,2]. Generated by facilities such as clinics, hospitals, dental offices, blood banks, animal hospitals, and medical research laboratories, this waste poses a substantial risk of infectious disease transmission and is classified as hazardous [3]. In Turkey, the management of medical waste is primarily governed by the “Medical Waste Control Regulation” (MWCR), which was published in the Official Gazette on January 25, 2017 [4]. This regulation delineates procedures and principles governing the entire lifecycle of medical waste, from its generation to final disposal. To assess treatment options for infectious waste and determine the most sustainable approach, the modified Sustainability Assessment of Technologies method is employed. Among the evaluated options, hydroclave emerges as the top performer across technical, economic, social, and environmental criteria, followed by autoclave, autoclave with a shredder, chemical treatment, demolizer, microwave, chem-clav, and central incineration Rahmani et al., 2020 [5]. In the context of medical waste management, the Polyclinic employs heat-based treatments such as autoclave, hydroclave, and microwave to decontaminate various materials, including sharps, syringes, and contaminated textiles [6].

In addressing the complexities of medical waste management, fuzzy logic serves as a tool to handle uncertainty and imprecise information, while multi-criteria decision-making (MCDM) incorporates multiple criteria into the decision-making process [[7], [8], [9], [10], [11], [12], [13]]. Fuzzy multi-criteria decision-making (F-MCDM) specifically addresses decision-making under uncertainty [14]. F-MCDM's application to real-world problems is advantageous, particularly for complex decisions involving uncertainty [[15], [16]]. Various studies have demonstrated the applicability of F-MCDM to real-world scenarios [[17], [18], [19], [20], [21], [22], [23], [24], [25], [26], [27], [28], [29], [30], [31], [32]]. In addressing complex issues like medical waste disposal, leveraging these methods to navigate uncertain scenarios is feasible. Thus, this article employs a multidimensional dataset and a hybrid MCDM approach, incorporating Fuzzy Preference Selection Index (F–PSI) and Fuzzy Compromise Ranking of Alternatives from Distance to Ideal Solution (F-CRADIS), to evaluate medical waste disposal methods.

### 1.1 Research gaps and novelties

A comprehensive examination of existing academic literature concerning waste disposal techniques reveals two critical research gaps. The first gap pertains to the absence of a universally accepted set of criteria for assessing and evaluating the selection of medical waste disposal methods. While several considerations have been proposed, including establishment costs, operational expenses, waste reduction efficiency, energy recovery rates, accessibility, business viability, social acceptance, and staffing requirements, there remains a need for a standardized framework. Despite researchers gravitating towards similar evaluation criteria, there exists a strong consensus regarding both quantitative and qualitative factors used in assessing waste disposal methods. However, these evaluation criteria may require further refinement to furnish comprehensive insights into the economic and technological aspects of disposal techniques and their contributions to sustainability, particularly from environmental and social perspectives.

The novelty of this study lies in its pioneering application of a comprehensive multi-criteria decision-making (MCDM) model to address the pressing issue of medical waste management exacerbated by the COVID-19 pandemic. It marks the first-time implementation of the Fuzzy Preference Selection Index (F–PSI) and Fuzzy Compromise Ranking of Alternatives from Distance to Ideal Solution (F-CRADIS) methods in real-life problem-solving within this context. By integrating economic, technological, environmental, and social considerations, the framework proposed in this study offers a holistic approach to selecting optimal medical waste treatment methods. The emphasis on disinfection efficiency as a pivotal criterion underscores the practical relevance and importance of the findings, with autoclaving identified as the preferred method. The validation of these strategies through a practical case study conducted in Sivas, Turkey, further enhances the credibility and applicability of the proposed approach. Multiple sensitivity analyses reinforce the robustness and reliability of the methodology, highlighting its potential impact in addressing critical challenges in medical waste management amidst the ongoing global health crisis.

The main contributions of the proposed approach can be summarized as follows:

• Proposal of a fuzzy decision-making approach based on fuzzy logic for evaluating medical waste disposal methods and selecting the best disposal method.
• Enablement of decision-makers to use linguistic terms in the evaluation process.
• Presentation of a hierarchical structure with a fuzzy methodological framework based on PSI and CRADIS methods for selecting medical waste disposal techniques.
• Conduct of a real-time case study in Sivas, which ranks 78th among Turkey's 81 provinces in population density, to validate the proposed frameworks.
• Corroboration of the focus of relevant authorities on using “autoclave” as a medical waste disposal technique for Sivas, supporting the study's findings.
• Performance of sensitivity analysis and comparison analysis to demonstrate the strength and consistency of the proposed MCDM framework. The findings confirm that the MCDM approach is relatively stable and consistent.

### 1.2 Research questions

The research questions of this article are presented below, aligned with the selection of the medical waste technique, previous studies, and the identified gaps in the literature.

Q1 Are there any decision support systems or mathematical tools applied to evaluate the choice of medical waste disposal technique?

Q2 What are the main criteria affecting the evaluation process in selecting the medical waste disposal technique?

Q3 Given a multidimensional dataset, which medical waste disposal technique is more successful?

As the selection of waste disposal techniques represents an optimization problem within the MCDM literature, addressing these critical research questions can aid various decision-makers in identifying a reliable, realistic, and applicable methodological framework.

The remainder of this work is organized as follows: section 2 provides a comprehensive literature review of studies utilizing components of the proposed model evaluation approach, section 3 elaborates on the procedures for F–PSI and F-CRADIS, section 4 presents a case study and the results of the proposed decision methodology and sensitivity analysis, section 5 summarizes the administrative, theoretical, and practical implications of the study. Finally, section 6 concludes the work, discusses the limitations of the proposed method, and offers recommendations for future research.

## 2 Literature review

The motivation for this study encompasses two essential objectives. Firstly, the primary aim is to propose a set of critical evaluation criteria for assessing medical waste disposal techniques. After reviewing similar articles in the literature, 16 evaluation criteria across four main dimensions were identified to compare medical waste disposal techniques. The criteria in the first dimension represent the environmental impact of the medical waste technique. The criteria in the second dimension pertain to the economic evaluation of medical waste techniques, encompassing technical information in the third dimension. Lastly, the selected criteria are based on social evaluation strategies, covering criteria that have yet to be explored in the literature. Conducting multidimensional performance analysis provides decision-makers with an alternative evaluation opportunity, supporting them in making more reasonable and consistent decisions regarding business processes and outcomes.

The second goal of this study is to develop a new evaluation methodology to analyze the choice of medical waste technique. For this purpose, a combination of Fuzzy Preference Selection Index (F–PSI) and Fuzzy Compromise Ranking of Alternatives from Distance to Ideal Solution (F-CRADIS) was developed, enabling the evaluation of medical waste disposal techniques in Sivas. The motivation for combining these MCDM tools lies in their respective advantages:

• The PSI procedure reduces subjectivity compared to other subjective weighting systems such as AHP and BWM, providing decision-makers with a straightforward and applicable mathematical algorithm regardless of the number of criteria in the model. It allows for broad flexibility in expressing decision-maker preferences and minimizes inconsistencies that may arise in models using pairwise comparison procedures.
• The CRADIS procedure offers a maximally stable and consistent MCDM framework, ensuring that changes in criteria or alternatives do not impact ranking results. It allows for a more flexible decision-making environment and provides a simple and applicable algorithm for decision-makers to make rational, consistent, and objective decisions.

Despite being a newly developed MCDM tool, the integration of this algorithm into the solution process of various decision problems in different fields demonstrates its significant advantages.

Additionally, the study aims to demonstrate the applicability of the proposed decision-making tool in selecting medical waste disposal techniques. Fuzzy methods, introduced by Zadeh in 1965, allow for the inclusion of qualitative values in the analysis, aligning more closely with human thought processes. This represents an advantage of fuzzy methods over simple MCDM methods. The study utilizes membership functions to convert qualitative values into fuzzy numbers, facilitating their use in fuzzy methods. Furthermore, the study presents the application of the proposed methodology by comparatively analyzing nine medical waste disposal techniques and 16 evaluation factors across four different dimensions for the province of Sivas. The literature review, along with the application of MCDM methods in waste disposal, provides a brief overview of studies where the F–PSI and F-CRADIS methods have been applied.

### 2.1 Application of MCDM methods in waste disposal

Previous studies have utilized various MCDM models to evaluate site selection [33,34], technique selection [35]; [36], and disposal management for healthcare facilities generating waste. Field research and personnel interviews have been common approaches in these studies. For instance, in landfill site selection studies, spatial analysis and Multi-Criteria Decision Making (MCDM) techniques have been integrated with Geographic Information Systems (GIS). Unal et al. [37] employed fuzzy Simple Multi-Attribute Rating Technique (SIMOS) to select disposal sites for solid waste in Adana, Turkey. Khorsandi et al. [38] utilized Analytic Hierarchy Process (AHP) and Technique for Order of Preference by Similarity to Ideal Solution (TOPSIS) for selecting regular storage spaces in Naqadeh, Iran. Chabok et al. [39] employed fuzzy AHP for selecting a solid waste landfill in Ahvaz, Iran. Zarin et al. [40] utilized fuzzy AHP for selecting locations for solid waste landfills in Islamabad, Pakistan. Ali et al. [41] conducted regular storage field selection for Memari, India, using AHP and fuzzy TOPSIS. Mishra and Rani [42] selected a disposal site for healthcare waste in Uttarakhand, India, using the Fermatean Fuzzy Weighted Aggregated Sum Product Assessment (WASPAS) method. Meng et al. [43] performed a two-stage optimal facility location selection for a waste-to-power plant using the Evaluation based on Distance from Average Solution (EDAS) method with single-valued neutrosophic sets. Beheshtinia et al. [44] introduced a new MCDM technique called PROMSIS for determining the location of a healthcare waste disposal center. These studies highlight the diverse methodologies employed to address the complex challenges associated with healthcare waste management and underscore the importance of employing suitable decision-making tools for effective waste management strategies.

Several studies have underscored the significance of selecting appropriate techniques for medical waste disposal. Belhadi et al. [45] utilized Analytic Hierarchy Process (AHP) and VIKOR methods for Prayagraj, India. Li et al. [46] employed Fuzzy Decision Making Trial and Evaluation Laboratory (DEMATEL) and Fuzzy Technique for Order of Preference by Similarity to Ideal Solution (TOPSIS) in Beijing, China. Abdelhafeez et al. [47] studied an enhanced version of Elimination and Choice Expressing Reality (ELECTRE) in neutrosophic media for waste disposal technique selection. Jaisankar et al. [48] utilized the Additive Ratio Assessment (ARAS) and ENTROPY methods in a probabilistic fuzzy set environment to select the optimal healthcare plastic waste disposal technique. Patel et al. [49] proposed an intuitionistic fuzzy version of the Elimination and Choice Translating Reality (EM) method followed by Step-wise Weight Assessment Ratio Analysis (SWARA) and TOPSIS to select the best medical waste treatment technique. Menekşe and Akdağ [50] extended the CRITIC-WASPAS method to single and interval-valued global fuzzy sets for selecting medical waste disposal alternatives. Çelik et al. [51] investigated waste management planning using Intuitive Fuzzy Weighted Averaging (IFWA), Intuitive Fuzzy Analytical Hierarchy Process (IFAHP), and Intuitive Fuzzy Technique for Order Preference by Similarity to Ideal Solution (IFTOPSIS). Görçün et al. [52] proposed the Interval-Valued Fermatean Fuzzy Delphi-SWARA-COPRAS model to address waste disposal company selection problems. Beheshtinia et al. [53] introduced a novel MCDM method called Fuzzy Relative Value Interval-based Compromise Ranking (RVIKOR) for healthcare waste disposal method selection. Kumar et al. [54] utilized the CRITIC and SWARA methods along with a new Pythagorean fuzzy similarity measure to determine the weightage of factors affecting the choice of a medical waste treatment method. Anafi et al. [55] applied the Criteria Importance Inter-criteria Correlation-Multi-Attribute Utility Theory (CRITIC-MAUT) model based on T-spherical fuzzy sets (T-SFS) for the selection of medical waste treatment technologies. These studies demonstrate the diverse methodologies and approaches employed to address the critical issue of medical waste disposal, emphasizing the importance of selecting suitable techniques for effective waste management strategies.

In addition, performance analyses in medical waste management were also carried out. Etim et al. [56] assessed Nigeria's strategies for managing medical waste in Ota with fuzzy AHP. Mardani et al. [57] evaluated the achievements of hospitals in Tehran, Iran, in medical waste management using fuzzy AHP. Rawal [58] measured hospitals' performance in Prayagraj, India, based on waste management practices using the AHP method. Unlike all these studies, Haseli and Ghoushchi [59] evaluated the best criteria to designate the plant's location to be installed in waste disposal with the fuzzy base criteria method for Tabriz, Iran. Puška et al. [60] decided on the type of incinerator used to dispose of health waste with the methods of FUCOM and CRADIS for Bosnia and Herzegovina.

Fuzzy logic is a method used to process uncertainty and imprecise information. Multi-criteria decision-making is a methodology that considers multiple criteria and manages the decision-making process based on these criteria. Fuzzy multi-criteria decision-making (F-MCDM) focuses on decision-making under uncertainty and uncertain information. Its adaptation to real-world problems can be beneficial, especially when complex decisions involving uncertainty are required. There are some examples showing that F-MCDM can be applied to real-world problems [[17], [18], [19], [20], [21], [22], [23],^26^,^27^]. In complex issues such as medical waste disposal, using these two methods to deal with uncertain situations may be possible. Based on what has already been said, this article uses a multidimensional dataset and a hybrid MCDM approach, which includes F–PSI and F-CRADIS, to look at the choice of medical waste disposal method.

### 2.2 Applications of F–PSI and F-CRADIS methods

In previous studies utilizing the Fuzzy Preference Selection Index (F–PSI) method: Ulutaş et al. [61] applied F–PSI to weigh criteria for selecting companies transporting ready-to-wear products for retailers in Turkey. Zhang et al. [62] enhanced the F–PSI method by developing a similarity-proximity weight-based determination approach to determine criterion weights in IVq-ROFN settings. Gangwar et al. [63] utilized F–PSI to select the most suitable alternative ship hull material based on the properties of aluminium metal alloy. Kundu et al. [64] employed F–PSI to select magnetic resonance imaging devices for private hospitals. Toslak et al. [65] utilized F–PSI, SV, and MARCOS methods for selecting peanut butter machines. Kumar et al. [66] evaluated wear parameters of ceramic coating using a fuzzy PSI-CODAS model. Regarding CRADIS and F-CRADIS: Puška et al. [60] employed CRADIS to select the optimal incinerator type for addressing medical waste in ancillary health facilities in Bosnia and Herzegovina. Puška et al. [67] conducted market assessment of pear variety produced in Serbia using F-CRADIS. Dordevic et al. [68] integrated linear programming with the MCDM model and rough CRADIS method for solving production optimization problems. Weizhong et al. [69] proposed the Fine-Kinney framework for CSFNs based on the extended CRADIS method. Krishankumar and Ecer [70] developed the q-step ortho-dual fuzzy CRADIS method for selecting IoT service providers for sustainable transport. Puška et al. [71] proposed the IMF SWARA-fuzzy CRADIS model for selecting distribution centre locations. Puška et al. [72] applied the IMF SWARA-Fuzzy TRUST CRADIS method for selecting sustainable suppliers. Biswas et al. [73] proposed the GC-CRADIS model for portfolio selection for investment in equities. Puška et al. [74] used the fuzzy LMAW-fuzzy ENTROPY-fuzzy CRADIS model for insurance company selection in agriculture. Wang et al. [75] developed the IVSF-Choquet integral-CRADIS model for risk prioritization in Fine-Kinney.

## 3 Research methodology

**Fig. 1:**
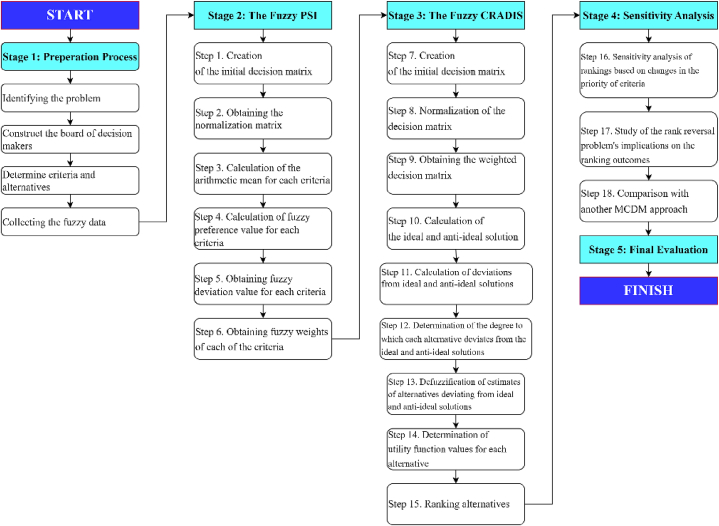
The flowchart of the F–PSI F-CRADIS multi-criteria model.

This article proposes an integrated model of Fuzzy Preference Selection Index (F–PSI) and Fuzzy Comprehensive Ranking and Decision Integration System (F-CRADIS) procedures to prioritize the criteria influencing the selection of medical waste disposal techniques and rank and evaluate these techniques. The flowchart of the proposed model for evaluating the choice of medical waste disposal technique is depicted in Fig. 1. Criteria were meticulously chosen based on previous research findings and expert opinions from the literature to structure the suggested decision-making model for identifying the best technique. A comprehensive set of 16 performance indicators was selected, spanning four dimensions to ensure a holistic assessment framework. These dimensions encompass a wide range of factors, including operational effectiveness, environmental impact, financial feasibility, and socio-ethical ramifications, each represented by specific, measurable indicators. Proceeding with the process, the F–PSI method, renowned for its robustness in handling uncertain and imprecise data, was employed to compute the weight values of each evaluation criterion. The F–PSI method adds a critical layer of precision and adaptability to the model by addressing the inherent vagueness in human judgments and the subjective nature of decision-making. Subsequently, after assigning weight values, the F-CRADIS technique was employed to establish a hierarchy among the alternatives. This method integrates the weighted criteria and ranks the potential techniques based on their overall performance, facilitating an informed and multifaceted decision-making process. The model's resilience to changes in input parameters was rigorously examined to ensure its ability to withstand variability, thereby solidifying its credibility as a decision-support tool in selecting the most viable technique.

### 3.1 Fuzzy theory

Zadeh [76] introduced fuzzy set theory to the literature as a means to represent uncertainty and enable decision-makers to assess using linguistic variables. Fuzzy numbers, which are specific subsets of fuzzy sets, play a crucial role in this framework. Fuzzy numbers can manifest in various forms in both theory and practice, serving as tools to express indefinite quantities. Among these, triangular fuzzy numbers are the most commonly utilized. Triangular fuzzy numbers have found widespread application in numerous studies for converting qualitative expressions into quantitative ones. Each triangular fuzzy number is characterized by three values: the lowest possible value, the most likely value, and the highest possible value. These values encapsulate the range of uncertainty associated with the fuzzy number, providing a means to quantify and analyze uncertain information effectively.

If $\tilde{A}=\left(a_{l},a_{m},a_{u}\right)$ and $\tilde{B}=\left(b_{l},{\;b}_{m},b_{u}\right)$ are two triangular fuzzy numbers, the mathematical computations related to these are defined in Eq. (1), Eq. (2), Eq. (3) and Eq. (4).

(1) $$\tilde{A}+\tilde{B}=\left(a_{l}+b_{l},a_{m}+b_{m},a_{u}+b_{u}\right)$$

(2) $$\tilde{A}-\tilde{B}=\left(a_{l}-b_{u},a_{m}-b_{m},a_{u}-b_{l}\right)$$

(3) $$\tilde{A}x\tilde{B}=\left(\min\left(a_{l}b_{l},a_{l}b_{u},a_{u}b_{l},a_{u}b_{u}\right),a_{m}b_{m},\max\left(a_{l}b_{l},a_{l}b_{u},a_{u}b_{l},a_{u}b_{u}\right)\right)$$

(4) $$\frac{\tilde{A}}{\tilde{B}}=\left(\min\left(\frac{a_{l}}{b_{l}},\frac{a_{l}}{b_{u}},\frac{a_{u}}{b_{l}},\frac{a_{u}}{b_{u}}\right),\frac{a_{m}}{b_{m}},\max\left(\frac{a_{l}}{b_{l}},\frac{a_{l}}{b_{u}},\frac{a_{u}}{b_{l}},\frac{a_{u}}{b_{u}}\right)\right)$$

Triangular fuzzy numbers could be transformed into crisp numbers with the help of different equations. For this study, Eq. (5) is employed to defuzzify a fuzzy number like $\tilde{A}=\left(a_{l},a_{m},a_{u}\right)$:

(5) $$A=\frac{a_{l}+4a_{m}+a_{u}}{6}$$

### 3.2 F– PSI method for prioritization of criteria

The following are main steps of F– PSI Method [61]:

Step 1 Creation of the initial decision matrix

DM utilizes the linguistic values from Table 1 to evaluate the criterion [77].

**Table 1:** Linguistic variables and fuzzy numbers.

Linguistic variables	Fuzzy numbers
Very high (VH)	(0.9, 1.0, 1.0)
High (H)	(0.7, 0.9, 1.0)
Medium-high (MH)	(0.5, 0.7, 0.9)
Medium (M)	(0.3, 0.5, 0.7)
Medium-low (ML)	(0.1, 0.3, 0.5)
Low (L)	(0.0, 0.1, 0.3)
Very low (VL)	(0.0, 0.0, 0.1)

The aggregate fuzzy decision matrix ($\tilde{Z}$) given in Eq. (6) is obtained using the geometric mean for all fuzzy judgments of DMs.

(6) $$\tilde{Z}={\left[{\tilde{z}}_{ij}\right]}_{kxn}=\left[\begin{array}{ccc} {\tilde{z}}_{11} & \cdots & {\tilde{z}}_{1n}\\ \vdots & \ddots & \vdots \\ {\tilde{z}}_{k1} & \cdots & {\tilde{z}}_{kn} \end{array}\right]$$

${\tilde{z}}_{ij}=\left(z_{ij}^{l},z_{ij}^{m},z_{ij}^{u}\right)$ in Eq. (6) is fuzzy value of criterion j. in alternative i.

Step 2 Obtaining the normalization matrix $\left(\tilde{T}\right)$.

Eq. (7) (for cost-specific criteria) and Eq. (8) (for benefit-specific criteria) are used to normalize the first decision matrix.

(7) $${\tilde{t}}_{ij}=\left(t_{ij}^{ı},t_{ij}^{m},t_{ij}^{u}\right)=\frac{\min\left({\tilde{z}}_{ij}\right)}{{\tilde{z}}_{ij}}=\left(\frac{\min\left(z_{ij}^{l}\right)}{z_{ij}^{u}},\frac{\min\left(z_{ij}^{l}\right)}{z_{ij}^{m}},\frac{\min\left(z_{ij}^{l}\right)}{z_{ij}^{l}}\right)$$

(8) $${\tilde{t}}_{ij}=\left(t_{ij}^{ı},t_{ij}^{m},t_{ij}^{u}\right)=\frac{{\tilde{z}}_{ij}}{\max\left({\tilde{z}}_{ij}\right)}=\left(\frac{z_{ij}^{l}}{{\max(z}_{ij}^{u})},\frac{z_{ij}^{m}}{{\max(z}_{ij}^{u})},\frac{z_{ij}^{u}}{{\max(z}_{ij}^{u})}\right)$$

Step 3 *Calculation of the arithmetic means*$\left({\bar{\tilde{t}}}_{ij}\right)$*for each criterion*.

With Eq. (9), the arithmetic means of the criteria are obtained.

(9) $${\bar{\tilde{t}}}_{ij}=\left(\bar{t_{ij}^{l}},\bar{t_{ij}^{m}},\bar{t_{ij}^{u}}\;\right)=\frac{\sum_{i=1}^{k}{\tilde{t}}_{ij}}{k}=\left(\frac{\sum_{i=1}^{k}t_{ij}^{l}}{k},\frac{\sum_{i=1}^{k}t_{ij}^{m}}{k},\frac{\sum_{i=1}^{k}t_{ij}^{u}}{k}\right)$$

Step 4 Calculation of fuzzy preference value $\left({\tilde{PV}}_{j}\right)$ for each criterion

Eq. (10) obtains the fuzzy preference value of the criteria.

(10) $${\tilde{PV}}_{j}=\left({PV}_{j}^{l},{PV}_{j}^{m},{PV}_{j}^{u}\right)=\sum_{i=1}^{k}{\left({\tilde{t}}_{ij}-{\bar{\tilde{t}}}_{ij}\right)}^{2}=\left(\min\left(\sum_{i=1}^{k}{\left(t_{ij}^{l}-\bar{t_{ij}^{l}}\right)}^{2},\sum_{i=1}^{k}{\left(t_{ij}^{u}-\bar{t_{ij}^{u}}\right)}^{2}\right),\sum_{i=1}^{k}{\left(t_{ij}^{m}-\bar{t_{ij}^{m}}\right)}^{2},\max\left(\sum_{i=1}^{k}{\left(t_{ij}^{l}-\bar{t_{ij}^{l}}\right)}^{2},\sum_{i=1}^{k}{\left(t_{ij}^{u}-\bar{t_{ij}^{u}}\right)}^{2}\right)\right)$$

Step 5 Obtaining fuzzy deviation value $\left({\tilde{\rho }}_{j}\right)$ for each criterion

With Eq. (11), fuzzy deviation values are obtained for each criterion.

(11) $${\tilde{\rho }}_{j}=\left(\rho_{j}^{l},\rho_{j}^{m},p_{j}^{u}\right)=\left|1-{\tilde{PV}}_{j}\right|=\left(\left|1-{PV}_{j}^{u}\right|,\left|1-{PV}_{j}^{m}\right|,\left|1-{PV}_{j}^{l}\right|\right)$$

Step 6 Obtaining fuzzy weights $\left({\tilde{w}}_{j}\right)$ of each of the criteria

By applying Eq. (12), the weights based on the importance coefficient of the criteria are calculated.

(12) $${\tilde{w}}_{j}=\left(w_{j}^{l},w_{j}^{m},w_{j}^{u}\right)=\frac{{\tilde{\rho }}_{j}}{\sum_{j=1}^{n}{\tilde{\rho }}_{j}}=\left(\frac{\rho_{j}^{l}}{\sum_{j=1}^{n}\rho_{j}^{u}},\frac{\rho_{j}^{m}}{\sum_{j=1}^{n}\rho_{j}^{m}},\frac{\rho_{j}^{u}}{\sum_{j=1}^{n}\rho_{j}^{l}}\right)$$

### 3.3 F-CRADIS method for sequencing alternatives

The following are the processes involved in the method's ordering of alternatives [67]:

Step 1 . Creation of the initial decision matrix (X).

DMs can assess the alternatives using the linguistic values listed in Table 2 [67].

Step 2 Normalization (N) of the decision matrix.

**Table 2:** Linguistic variables and fuzzy numbers.

Linguistic variables	Fuzzy numbers
Very high (VH)	(9, 10, 10)
High (H)	(7, 9, 10)
Medium-high (MH)	(5, 7, 9)
Medium (M)	(3, 5, 7)
Medium-low (ML)	(1, 3, 5)
Low (L)	(0, 1, 3)
Very low (VL)	(0, 0, 1)

If the criterion is benefit-oriented, normalization is carried out with Eq. (13), and if the criterion is cost-oriented, normalization is carried out with Eq. (14).

(13) $$\tilde{n}=\left(n_{ij}^{l},n_{ij}^{m},n_{ij}^{u}\right)=\left(\frac{x_{id}^{l}}{x_{ij}^{u}},\frac{x_{id}^{l}}{x_{ij}^{m}},\frac{x_{id}^{l}}{x_{ij}^{l}}\right)$$

(14) $$\tilde{n}=\left(n_{ij}^{l},n_{ij}^{m},n_{ij}^{u}\right)=\left(\frac{x_{ij}^{l}}{x_{id}^{u}},\frac{x_{ij}^{m}}{x_{id}^{u}},\frac{x_{ij}^{u}}{x_{id}^{u}}\right)$$

Step 3 Obtaining the weighted decision matrix (V).

Eq. (15) is used to construct the aggravated decision matrix. To create it, multiply the appropriate weight by the value of the normalised decision matrix.

(15) $${\tilde{v}}_{ij}=\left(v_{ij}^{l},v_{ij}^{m},v_{ij}^{u}\right)={\tilde{n}}_{j}*{\tilde{w}}_{j}$$

Step 4 Calculation of the ideal and anti-ideal solution

The weighted decision matrix's highest value, $v_{ij}$, put to use to calculate the ideal solution, while the weighted decision matrix's lowest value, $v_{ij}$, put to use to calculate the anti-ideal solution. It is obtained by Eq. (16) and Eq. (17), respectively.

(16) $$t_{i}=\max\;{\tilde{v}}_{ij}$$

(17) $$t_{ai}=\min\;{\tilde{v}}_{ij}$$

Step 5 Calculation of deviations from ideal and anti-ideal solutions

Deviations from the ideal and anti-ideal solutions are adopted by using Eq. (18) and Eq. (19), respectively.

(18) $$d^{+}=t_{i}-{\tilde{v}}_{ij}$$

(19) $$d^{-}={\tilde{v}}_{ij}\;-t_{ai}$$

Step 6 Determination of the degree to which each alternative deviates from the ideal and anti-ideal solutions

The degree to which the alternatives deviate from the ideal and anti-ideal solutions is adopted by Eq. (20) and Eq. (21).

(20) $$s_{i}^{+}=\sum_{j=1}^{n}d^{+}$$

(21) $$s_{i}^{-}=\sum_{j=1}^{n}d^{-}$$

Step 7 Defuzzification of estimates of alternatives deviating from ideal and non-ideal solutions

The degree of deviation from the ideal and anti-ideal solutions calculated for each alternative is defuzzification by Eq. (22).

(22) $$s_{i_{\mathrm{def}}}^{\pm }=\frac{d_{i}^{l}+4d_{i}^{m}+d_{i}^{u}}{6}$$

Step 8 Determination of utility function values for each alternative.

Obtained using Eq. (23) and Eq. (24).

(23) $$K_{i}^{+}=\frac{s_{0}^{+}}{s_{i}^{+}}$$

(24) $$K_{i}^{-}=\frac{s_{i}^{-}}{s_{0}^{-}}$$

The optimum alternative closest to the ideal solution is $s_{0}^{+}$, whereas the optimal alternative farthest from the anti-ideal solution is $s_{0}^{-}$.

Step 9 Ranking alternatives

Eq. (25) average departure of the options from the degree of utility yields the final ranking.

(25) $$Q_{i}=\frac{K_{i}^{+}+K_{i}^{-}}{2}$$

The option with the highest $Q_{i}$ value is the best alternative.

## 4 Case study

A case study from Sivas, in the middle of Turkey, was included to show how the suggested method for removing medical waste could be used. Sivas is the second-biggest city in Turkey after Konya, with a total area of 28.488 km^2^. It is positioned between 36° and 39° degrees east longitude and 38°and 41° degrees north latitude. Sivas has a population density that places it 78th out of 81 provinces, according to the Address-Based Population Registration System Results for 2021 from the Turkish Statistical Institute (TurkStat). Table 3 lists the quantity of medical waste generated in Sivas before COVID-19 and as of the present.

**Table 3:** Medical waste amount in Sivas over the years [78].

Years	Medical Waste Amount (tons)
**2016**	780
**2017**	830
**2018**	878
**2019**	855
**2020**	1141
**2021**	1049
**2022**	1104
**2023 (first nine months)**	756

**Fig. 2:**
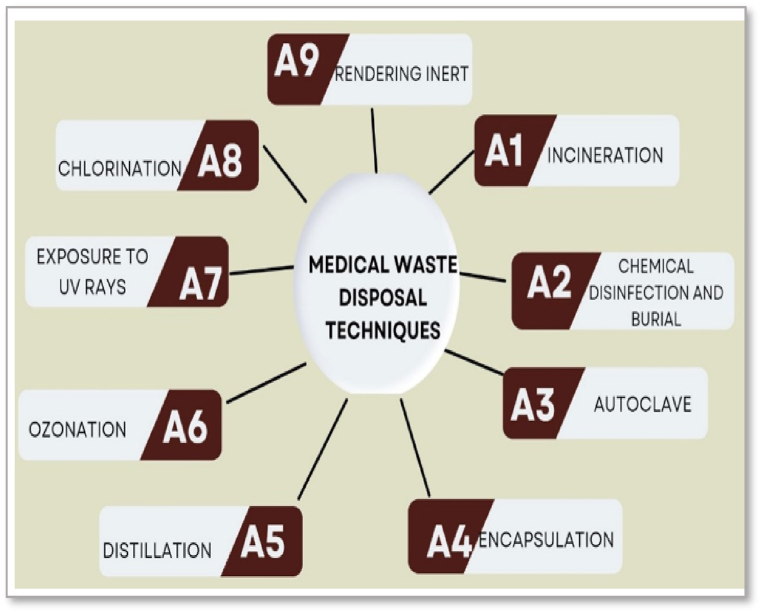
The nine medical waste disposal alternatives for case study.

According to the data in Table 3, the amount of medical waste has increased with the increase in COVID-19 cases in the province. In Sivas, which disposes of these wastes, there is a medical waste disposal facility with a history of 10 years, located 17 km from the settlements and 1 km from the landfill of the SM. Fig. 2 presents an array of medical waste disposal alternatives, each with its own definition and description. This diversity in options showcases a spectrum of technologies tailored to address the complexities associated with medical waste management. Belhadi et al. [45], Chisholm et al. [79], Manupati et al. (2021).

• A1: Incineration: Incineration is a widely used method characterised by high-temperature waste destruction using various techniques. Furnace burning, rotary ovens, and pyrolytic furnace incineration leverage the combustion process. Pyrolysis and gasification are advanced techniques within this category that decompose organic waste materials by heating in the absence of oxygen, thereby minimising toxic emissions.
• A2: Chemical Disinfection and Embedding This alternative involves using chemicals to neutralise or destroy pathogens in medical waste. The process ensures that infectious agents are rendered harmless before the waste is safely disposed of or handled further.
• A3: Autoclave: Autoclaving uses high-pressure steam at elevated temperatures to sterilise waste. It is widespread for wastes that can withstand moisture and heat, making it an efficient method for many medical waste types.
• A4: Encapsulation: This method ensures that the waste is securely contained within containers using a binding substance. Isolating the waste prevents the leaching of contaminants and direct contact with the environment or handlers.
• A5: Distillation: Used predominantly for liquid wastes, distillation separates mixtures based on differences in their volatilities in a boiling liquid mixture. It is effective for recycling certain chemicals but less for complex medical waste management.
• A6: Ozonation: Ozonation employs ozone, a strong oxidant, to inactivate microorganisms in the waste. It is beneficial for water purification in waste treatment facilities and can also be applied to solid waste.
• A7: UV Ray Exposure: Ultraviolet radiation can effectively destroy pathogens such as viruses and bacteria, making UV rays an excellent non-chemical disinfection method for surface-level contamination.
• A8: Chlorination: Chlorination is traditionally reserved for water treatment, effectively disinfecting wastewater by killing bacteria and viruses using chlorine or chlorine compounds.
• A9: Rendering Inert: Rendering waste inert is a process suited for the more challenging types of medical waste, like those with high pharmacological content or heavy metals. These are often stabilised with cement or other substances to prevent the leaching of toxins, thereby safeguarding against environmental contamination.

An expert group of 5 DMs (DM1, DM2, DM3, DM4 and DM5) was formed to determine the most preferred medical waste disposal technique. The information of 5 DMs is given in Table 4.

**Table 4:** Properties of DMs.

Decision-Maker	Duty	Experience	Workspace
**DM1**	Administrator	17	Zero waste and climate change
**DM2**	Environmental engineer	10	Climate change
**DM3**	Environmental engineer	9	Waste and recycling
**DM4**	Expert biologist	15	Medical waste
**DM5**	Field expertise	13	All waste

**Fig. 3:**
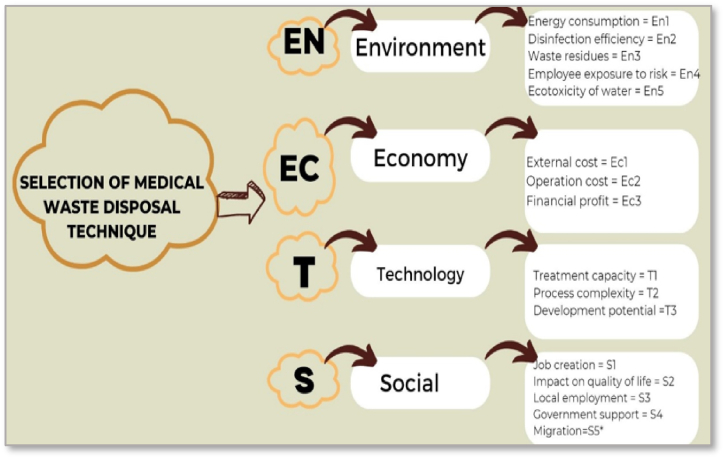
The 16 criteria that affect medical waste disposal techniques selection.

The five selected DMs were surveyed and recorded for their comprehensive knowledge of medical waste disposal. Alternatives were evaluated by considering the criteria. DMs evaluated several methods of eliminating medical waste by employing linguistic factors and significance weights associated with the pertinent criteria. Four dimensions and a total of 16 criteria that affect the selection of medical waste disposal techniques were used. These criteria are used in terms of environment, economy, technology, and social dimensions, and a concise definition of these criteria is shown in Fig. 3 [[80], [81], [82]].

The Environment (En) Dimension assesses the environmental impact of medical waste treatment methods.

• Energy Consumption (En1): Measures the total energy requirements for treating a unit of waste, encompassing both the direct operational energy and the indirect energy attributed to the entire treatment lifecycle.
• Disinfection Efficiency (En2): Evaluate waste treatment methods' effectiveness in reducing pathogen concentrations in bacteria and viruses before and after disinfection.
• Waste Residues (En3): Quantifies air pollution in terms of CO2 (g) equivalent emissions produced during waste treatment.
• Employees' Exposure to Risk (En4): Assesses the potential risk employees might encounter while managing and handling infectious waste.
• Ecotoxicity of Water (En5): Measures the harmful substances discharged into groundwater due to waste treatment processes over a specified period.

Economic (EC) Dimension examines the financial aspects:

• External Cost (Ec1): Cost associated with environmental emissions, worker health and safety, and ecological corrections due to land use.
• Operation Cost (Ec2): Includes the entire costs associated with running waste treatment facilities, like supplies, energy, services, and personnel.
• Financial Profit (Ec3): Gains realised from selling by-products such as fertilisers, energy, and recyclable materials or savings from the reduced necessity to dispose of waste.

Technology (T) Dimension is focusing on the technical parameters:

• Treatment Capacity (T1): The volume of waste that can be processed within a given timeframe.
• Process Complexity (T2 and T3): These criteria seem to measure the operational difficulties caused by the tasks, workflows, and decisions that need to be made up to the output stage, but they are actually defined in two different but mistakenly identical ways. T3 also aims to evaluate potential technological advancements; however, it is stated the same as T2 and might require clarification or amendment.

Social (S) Dimension addresses the societal impacts:

• Job Creation (S1): The total number of employment hours generated during the construction and operational phases of the waste treatment installation.
• Impact on Quality of Life (S2): the measure of how waste treatment solutions affect citizens' daily lives, including factors like odour and noise.
• Local Employment (S3): The degree to which the local workforce and resources are engaged in developing and operating the treatment processes.
• Government Support (S4): The extent of backing by governmental and municipal bodies for the waste treatment process.
• Migration (S5): This novel criterion, introduced against the backdrop of population shifts observed during COVID-19, examines the influence of waste treatment processes on population distribution, specifically the movement from densely to less densely populated areas.

*Note*: The criterion “Migration (S5)” is highlighted as a new inclusion within the literature and was added to address specific socio-economic shifts experienced in Sivas during the COVID-19 pandemic as per the request of the stakeholders in the study (SM).

Articulating these dimensions and criteria reflects an effort to construct a comprehensive and multidimensional approach to assessing waste disposal alternatives. Integrating environmental, economic, technological, and social factors provides a holistic framework to guide decision-making in the sustainable treatment and disposal of medical waste. The emphasis on diverse criteria underlines the complexity of waste management and the need for multi-criteria decision analysis in this area.

## 5 The adoped results

The evaluation of dimensions according to each alternative was performed by 5 DMs with a fuzzy scale presented in Table 1. DMs individually assessed the performance of the dimensions. Using Eqs. (7), (8), (9), (10), (11), (12), fuzzy PSI results for dimensions were determined. The weights calculated using the F–PSI method determined that the environment was the most important dimension. In addition, the technology and economy dimensions are other critical dimensions.

Similarly, using Eqs. (7), (8), (9), (10), (11), (12). Then, the prioritization coefficients of the criteria calculated using F–PSI were normalised.

$$w_{j}={\left(\begin{array}{c} 0,0661\;0,0743\;0,0593\;0,0656\;0,0581\;0,0617\;0,0616\;0,0426\;\\ 0,0475\;0,0643\;0,0615\;0,0697\;0,0575\;0,0730\;0,0646\;0,0725 \end{array}\right)}^{T}$$

The weights calculated according to the F–PSI method determined that the disinfection efficiency shown by (En2) was the best criterion. Moreover, local employment (S3) and migration (S5) are other critical criteria.

Five DM evaluated the alternatives against each criterion using the fuzzy scale shared in Table 2. The geometric mean and these matrices were combined to create the first-choice matrix. Eq. (13) and Eq. (14) were used to normalize this matrix. Eq. (15) is used to weight the normalised fuzzy decision matrix once the data have been normalised.

Identifying ideal and anti-ideal solutions is required once the data have been normalised and weighted. The highest value of the weighted data (max. 0.1099) represents the ideal solution, while the lowest value of the same data represents the anti-ideal solution (0.0013). Deviations from ideal and anti-ideal solutions were calculated by Eq. (18) and Eq. (19), and the sum of these deviations was obtained by Eq. (20) and Eq. (21). With Eq. (22), these degrees of deviation are defuzzified. Each alternative's utility function is identified using Eq. (23) and Eq. (24) after being obtained via Eq. (25).

### 5.1 Sensitivity analysis of rankings based on changes in the priority of criteria

An extensive sensitivity analysis that consists of three stages is performed in this section to validate the results of the suggested methodology. In this context, firstly, the impacts of modifying the criteria weights on the ranking results are investigated. Secondly, the effects of the rank reversal problem on the ranking results are analyzed. Third and lastly, the proposed hybrid model is compared with other MCDM methods.

**Fig. 4:**
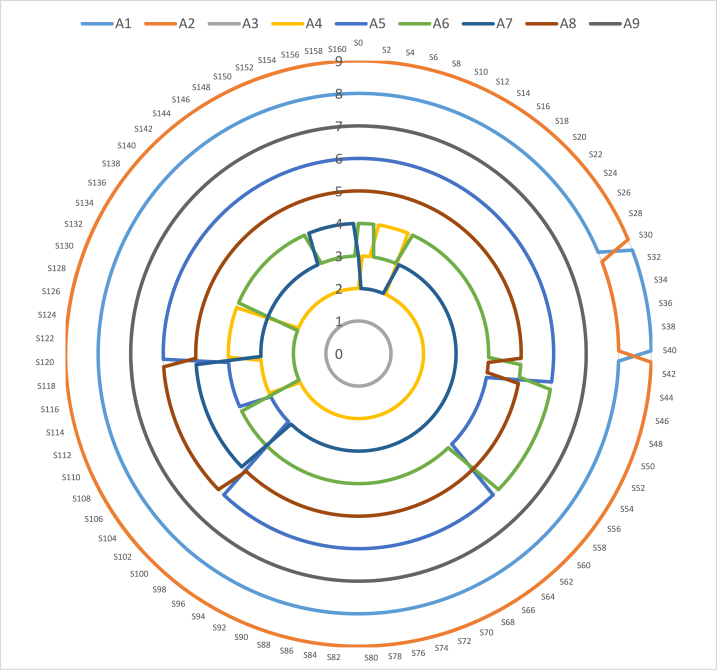
Ranking of alternatives based on the new criterion weights.

One hundred sixty different “scenarios” (S) were made to see if the proposed model's first result was stable and reliable (Gorcün et al., 2022). Regardless of which criterion is the most important, starting from the first criterion, the new values of each criterion are determined by decreasing the previous values of the respective criteria by 10%, 20%, …, and 100%, respectively, in each case. For example, in the first case, the new value of the first criterion was calculated as 0,0595. It was then proportionally added to each remaining criterion [(1–0.0595)/(16-1)], so long as the total weights for all the criteria equaled 1. After looking at the results from the 160 scenarios shown in Fig. 4, we found that option A3 stayed the best choice in all surveys, just like it was in the first one. Also, option A9 stayed in the same position, ranking 7th, in all surveys. We did a sensitivity check by adjusting the importance of each factor.

Other alternatives' ranking results for the sensitivity analysis, which involves changing the criteria weights, are also slightly different. However, these changes do not significantly influence overall results, confirming that the proposed evaluation approach is consistent and stable on a large scale.

### 5.2 Study of the rank reversal problem's implications on the ranking outcomes

In this study, we produced 7 different S, deleting the worst alternative in each case to test whether the proposed methodology was robust to the rank reversal problem [83]. The last alternative in each case is deleted, and changes in the ranking results are observed. As a result, the new ranking results show that A3 remains the best alternative in all S. This outcome, which displays that the proposed model resists the rank reversal problem, supports that the outcome from the first sequence is consistent and stable.

### 5.3 Comparison with another MCDM approach

**Fig. 5:**
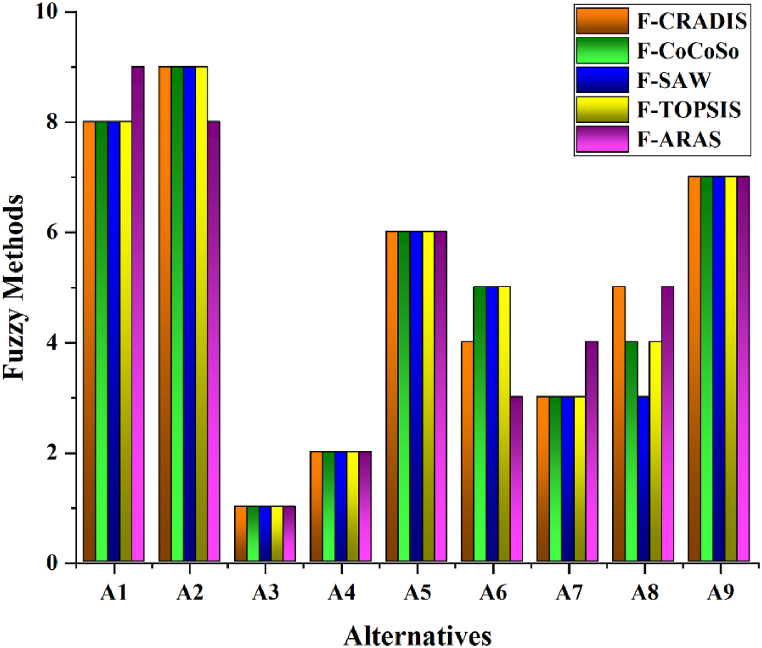
Comparison between ranking performances of MCDM methods.

Our study compares F-CRADIS results with those of other methods such as F-CoCoSo, F-SAW, F-TOPSIS, and F-ARAS. In Fig. 5, which shows the comparison results, the results are very consistent, even though the order of the alternatives ranked other than A3, A4, A5, and A9 has changed for all the MCDM methods that were used.

Last but not least, the statistical correlation of ranks using Spearman's correlation coefficient supports the suggested model. To determine the Spearman correlation coefficients, SPSS 28 was utilized. The proposed model's ranking with an average correlation value of 0,976 between the other four MCDM methods and the F-CRADIS approach can be confirmed as reliable.

## 6 Conclusions, limitations, and future directions

The current study proposes a new hybrid decision-making tool consisting of F–PSI and F-CRADIS to evaluate medical waste disposal techniques. The F–PSI approach is used to calculate the weights of waste disposal technique performance indicators using the hybrid model for performance analysis that has been provided. Using F-CRADIS, medical waste disposal methods are categorised and assessed.

The study is innovative in developing a unique, multifaceted approach to waste management by crafting a model that integrates various critical dimensions: economic, environmental, technological, and social. This model enables comprehensive analyses of waste management systems and diverges from traditional one-dimensional assessments. With a strong emphasis on safety and public health, the study underscores the significance of disinfection effectiveness and risk mitigation in managing medical waste. Practicality is demonstrated through case studies that align theoretical models with real-world scenarios, bridging the gap between research and practice. Additionally, the flexible structure of the study anticipates future changes in the waste management sector, allowing for adaptability and ongoing refinement. Collectively, these aspects of the research promise to significantly advance waste management practices and serve as a valuable tool for professionals, policymakers, and academics in the field.

Following the weighting results, the most critical dimension is the Environment. Other important dimensions are listed: Technology > Economy > Social. Again, following the weighting results, the most important criterion is disinfection efficiency (En2). Other criteria are listed as follows in order of importance: Local employment (S3) > Migration (S5) > Job creation (S1) > Energy consumption (En1) > Employees risk exposure (En4) > Government support (S4) > Process complexity (T2) > External cost (Ec1) > Operation cost (Ec2) > Development potential (T3) > Waste residues (En3) > Ecotoxicity of water (En5) > Impact on quality of life (S2) > Treatment capacity (T1) > Financial profit (Ec3). The ranking results from the introduced methodology show that A3 (autoclave) is the most preferred medical waste disposal technique. Other medical waste disposal techniques are listed as follows: A4 (encapsulation) > A7 (UV ray exposure) > A6 (ozonation) > A8 (chlorination) > A5 (distillation) > A9 (rendering inert) > A1 (incineration) > A2 (chemical disinfection and embedding).

We conduct a comprehensive sensitivity analysis of three stages to demonstrate the stability and feasibility of the proposed decision-making framework. In this context, the effect of changes in criterion weights on the first ranking is tested first. Secondly, the effect of the rank reversal problem is investigated. Finally, the sequencing results of the proposed methodology were compared with the F-TOPSIS, F-SAW, F-CoCoSo, and F-ARAS techniques. Findings from the sensitivity analyses show that the integrated model introduced provides maximum consistent and stable results.

This article introduces a decision-making model based on fuzzy theory that can effectively address the issue of choosing a method for disposing of medical waste. This study's focus is restricted to examining the selection of waste management methods within the geographical region of Sivas Province, located in Turkey. The study is limited to the criteria and alternatives used. Hence, it is not appropriate to extrapolate our findings to alternative methods of waste management in Turkey. For future novelties, one can broaden the research horizon by incorporating governance and machine learning into the criteria.

Also, the research topic could be looked into further in the future using different MCDM methods that use different fuzzy sets, such as the Fermatean fuzzy, the hesitant fuzzy, the intuitive fuzzy, the neutrophic fuzzy, the picture fuzzy, the Pythagorean fuzzy, the q-rung orthopair fuzzy, and the spherical fuzzy sets. This study provides a flexible strategy that can adapt to future variability. This is a crucial step to make future projections in the waste management field and be prepared for possible scenarios. Showing how the proposed model and methods can be applied in different geographical areas or health facilities emphasises that this approach is scalable and has the potential for a broad impact. The frameworks defined in Moslem [84] can also be generalised using the developed idea. In addition, the proposed model can be an efficient tool to estimate citizens' demand for addressing other related problems.

## Data availability statement

Data will be made available on request.

## Contributions

All authors contributed equally in preparing this manuscript.

## Ethical approval

This article does not contain any studies with human participants or animals performed by the author.

## CRediT authorship contribution statement

**Ahmet Turan Demir:** Writing – original draft, Methodology, Formal analysis, Data curation, Conceptualization. **Sarbast Moslem:** Writing – review & editing, Supervision, Project administration, Conceptualization.

## Declaration of competing interest

The authors declare that they have no known competing financial interests or personal relationships that could have appeared to influence the work reported in this paper.
